# ﻿First record of functional underground traps in a pitcher plant: *Nepenthespudica* (Nepenthaceae), a new species from North Kalimantan, Borneo

**DOI:** 10.3897/phytokeys.201.82872

**Published:** 2022-06-23

**Authors:** Martin Dančák, Ľuboš Majeský, Václav Čermák, Michal R. Golos, Bartosz J. Płachno, Wewin Tjiasmanto

**Affiliations:** 1 Department of Ecology and Environmental Sciences, Faculty of Science, Palacký University Olomouc, Šlechtitelů 27, CZ-78371 Olomouc, Czech Republic; 2 Department of Botany, Faculty of Science, Palacký University Olomouc, Šlechtitelů 27, CZ-78371 Olomouc, Czech Republic; 3 Department of Forest Protection and Wildlife Management, Faculty of Forestry and Wood Technology, Mendel University in Brno, Zemědělská 3, 613 00 Brno, Czech Republic; 4 School of Biological Sciences, University of Bristol, 24 Tyndall Avenue, Bristol, BS8 1TQ, UK; 5 Department of Plant Cytology and Embryology, Faculty of Biology, Institute of Botany, Jagiellonian University in Kraków, 9 Gronostajowa St., 30-387 Kraków, Poland; 6 Yayasan Konservasi Biota Lahan Basah, Jalan Raya Sawo 17–19, Surabaya, Indonesia

**Keywords:** Borneo, carnivorous plant, Caryophyllales, Mentarang Hulu, prey composition, taxonomy, underground trap

## Abstract

*Nepenthespudica*, a new species from North Kalimantan, Indonesia, is described and illustrated. The species belongs to the *N.hirsuta* group (sensu Cheek and Jebb 1999) but exhibits some characters that are unique within the group or even within the genus. Above all, it produces underground, achlorophyllous shoots with well-developed, ventricose lower pitchers that form in soil cavities or directly in the soil. No lower pitchers are formed above ground. The main part of its prey are ants, besides other litter- and soil-inhabiting species of invertebrates. A number of infaunal species were found in both aerial and underground pitchers, mainly Diptera and nematodes. *Nepenthespudica* is known only from a few neighbouring localities in the Mentarang Hulu district of North Kalimantan, where it grows on ridgetops at an elevation of 1100–1300 m. Its discovery underlines the natural richness of Borneo’s rainforest and the necessity to preserve this important ecosystem with its enormous and still undiscovered biodiversity.

## ﻿Introduction

*Nepenthes* L. is a genus of more than 160 species (Golos et al. 2020) primarily distributed in tropical and subtropical Southeast Asia, with centres of diversity in Borneo, Sumatra, and the Philippines. A small number of species occur in outlying areas, including Madagascar, Seychelles, Sri Lanka, northeastern India, southern China, northeastern Australia, and various islands of the western Pacific Ocean (McPherson et al. 2009). The *Nepenthes* flora of Borneo, with around 40 recognised species, is one of the most species-rich of all. Although the island is still partially covered with extensive primary forest, its area has been rapidly decreasing in recent decades (Miettinen et al. 2011). Commercial logging and subsequent land conversion (mostly for oil palm plantations) drastically reduced the area of pristine old-growth forest from 55.8 Mha in 1973 to 20.6 Mha in 2015 (Gaveau et al. 2016), making the Borneo rainforest one of the most rapidly vanishing ecosystems in the world. The island is botanically relatively well explored in the northern part, i.e. Malaysian Borneo (Sarawak and Sabah) and Brunei, where only remnants of untouched rainforest exist, usually protected as national parks and reserves. In contrast, Indonesian Borneo (Kalimantan) is one of the world’s least explored and most threatened biodiversity hotspots, still with vast areas of relatively intact forest (Raes et al. 2009). However, besides the expansion of oil palm plantations, the announced establishment of the new capital of Indonesia, Nusantara, in East Kalimantan might have a serious impact on the vulnerable biota of Borneo (e.g. Teo et al. 2020). The *Nepenthes* flora of Kalimantan is poorly known compared to that of Malaysian and Bruneian Borneo, with relatively few modern records. Thus, the new discoveries that have emerged recently after expeditions to certain remote areas of Kalimantan (Robinson et al. 2019; Golos et al. 2020) are not surprising.

Here we describe a new species of *Nepenthes* from lower montane rainforest in North Kalimantan, Indonesia, which produces well-developed, fully functional and effective underground traps – a strategy as yet unknown in any species of carnivorous plant with pitfall traps. While the majority of carnivorous plants produce their traps above ground or in water, underground traps have up till now been recorded only in the genera *Genlisea* Benth. & Hook.f., *Philcoxia* P.Taylor & V.C.Souza and *Utricularia* L. These genera use three different trapping mechanisms. While *Utricularia* employs actively working sucking utricles (i.e. Poppinga et al. 2016), *Genlisea* employs passive ‘lobster-pot’ type traps (Taylor 1991; Płachno et al. 2008). The adhesive leaves of *Philcoxia* are shallowly buried in sand to receive just enough light to maintain their photosynthetic ability (Pereira et al. 2012). On the other hand, pitfall traps (i.e. traps that rely on gravity) produced from wholly subterranean shoots that have evolved specifically to function underground have not been recorded in carnivorous plants so far (see, e.g. Darnowski et al. 2018).

## ﻿Materials and methods

This study is based on plants found in February 2012 in the Mentarang Hulu district of North Kalimantan province, Indonesia. A total of 17 plants were examined across five different sites. Plants were photographed, sampled and subsequently thoroughly compared with original drawings and descriptions given in protologues of morphologically allied *Nepenthes* species. Specimens of the *Nepentheshirsuta* group were examined in the herbaria BO, K and L (see Suppl. material 1) and the type material was deposited in BO (herbarium codes according to Thiers 2022).

For scanning electron microscopy (SEM), the representative trap parts were fixed in ethanol and later dehydrated and subjected to critical-point drying using liquid CO_2_. They were then sputter-coated with gold and examined at an accelerating voltage of 20 kV using a Hitachi S-4700 SEM (Hitachi, Tokyo, Japan), which is housed in the Institute of Geological Sciences, Jagiellonian University in Kraków.

Material for prey investigation was sampled from both underground (tree-root cavities) and aboveground pitchers. The entire contents of five lower pitchers and one aerial rosette pitcher was poured out through a 25 μm sieve, immediately fixed in 4% formaldehyde at circa 80 °C, and stored for 14–21 days, before insects and acarids including also larvae were separated and fixed again. The fine content including nematodes, annelids and organic detritus was transferred into glycerine according to De Grisse (1969) and finally mounted onto wax-glycerine slides and examined. Fixed specimens were identified under a light microscope and documented. All individuals that showed signs of digestion were considered prey. Individuals without signs of digestion were identified and assessed as either prey or infauna based on their biology and present life stages (e.g. larvae were mostly considered infauna). All insect and mite preparations are deposited at the Department of Entomology of Moravian Museum Brno. All the nematodes are deposited in the Department of Forest Protection and Wildlife Management in Brno. Permanent slides of *Pristinaarmata* (Naididae) are deposited at the National Museum in Prague, Czech Republic (Schenková and Čermák 2013).

## ﻿Taxonomic treatment

### ﻿ Nepenthes pudica

Taxon classificationPlantaeCaryophyllalesNepenthaceae

Dančák & Majeský sp. nov.

FC85A024-B0C6-5506-A71B-081479ADBEC1

urn:lsid:ipni.org:names:77300236-1

**Figure 1.**
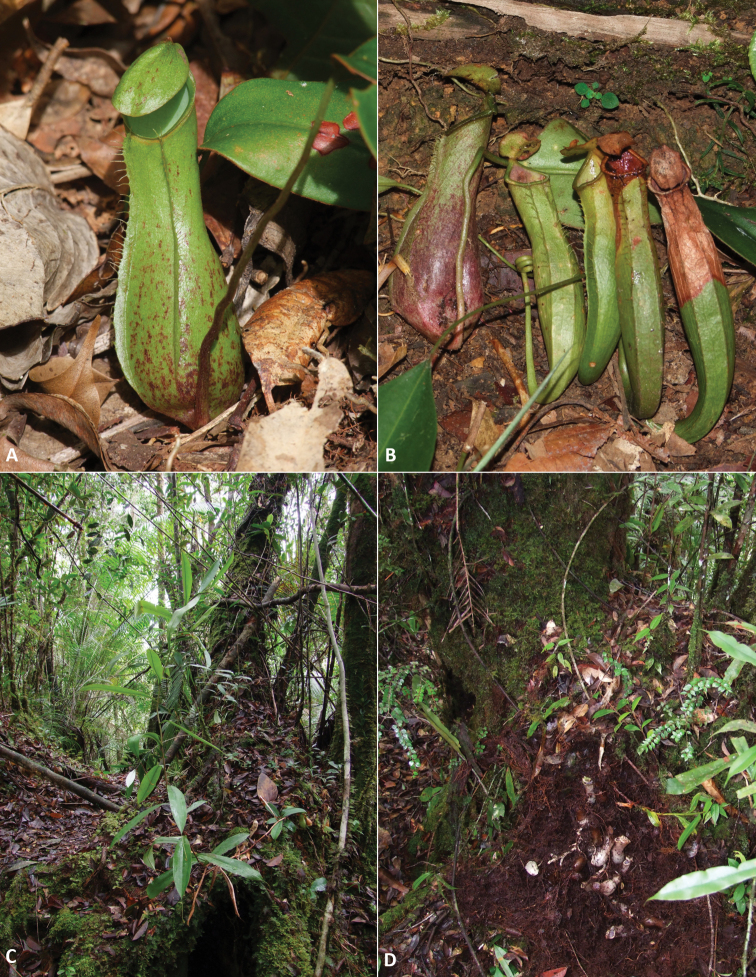
*Nepenthespudica***A** juvenile rosette pitcher **B** upper pitchers (4 on the right; each from a different plant), intermediate pitcher (1 on the left) **C** habitat with mature plant **D** habitat with lower pitchers excavated from the soil. Photographs by M. Dančák.

**Figure 2.**
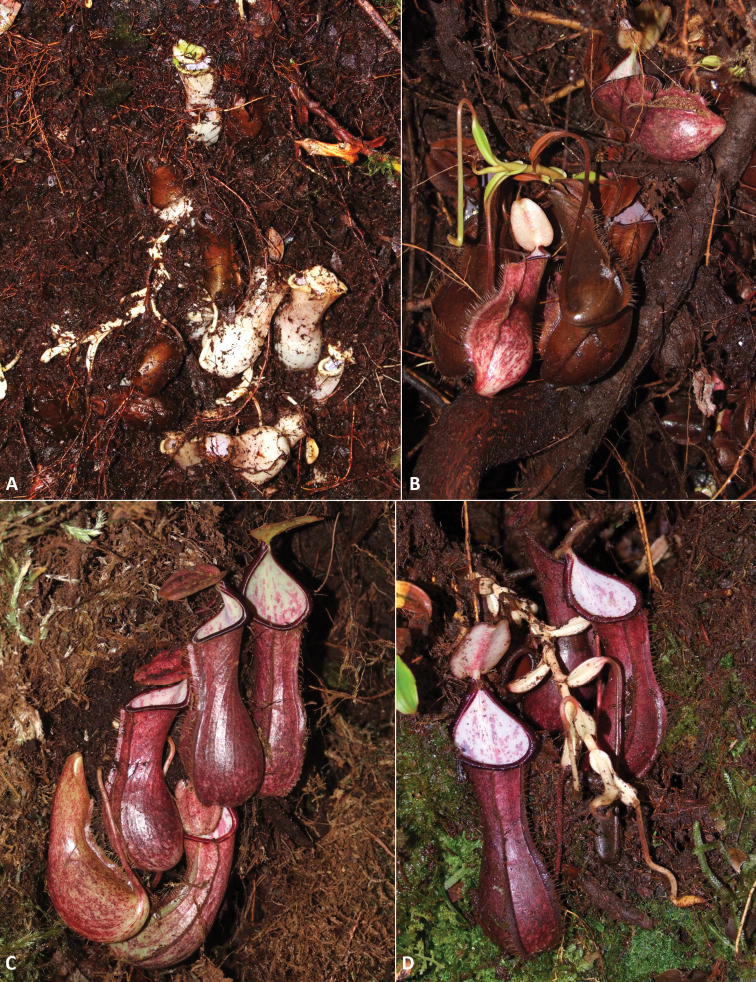
*Nepenthespudica***A** detail of lower pitchers excavated from the soil **B** lower pitchers in a cavity under tree roots–note greening of phyllodia formed in presence of low light **C** lower pitchers revealed under a moss mat **D** lower pitchers extracted from a cavity–note achlorophyllous shoot and reduced phyllodia formed in total darkness. Photographs by M. Dančák.

**Figure 3.**
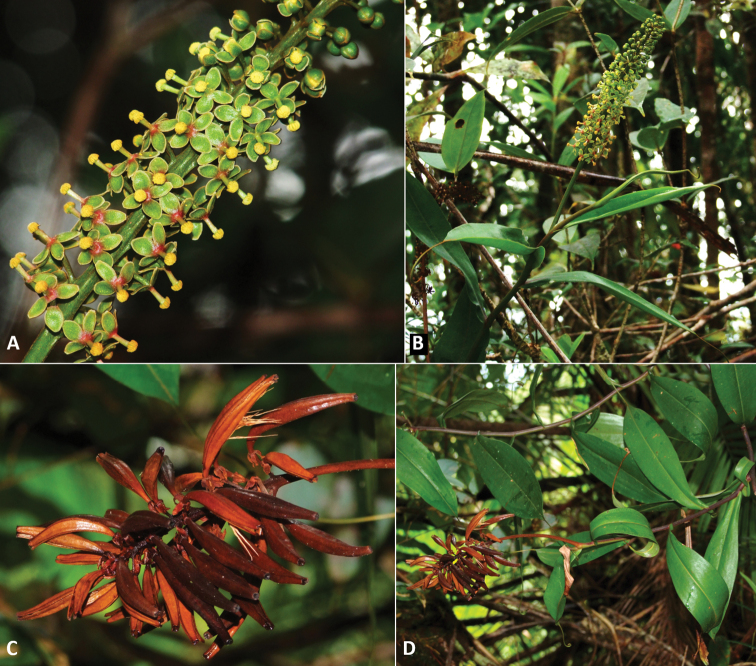
*Nepenthespudica***A** male flowers **B** male plant with inflorescence **C** infructescence **D** female plant with infructescence. Photographs by M. Dančák.

**Figure 4.**
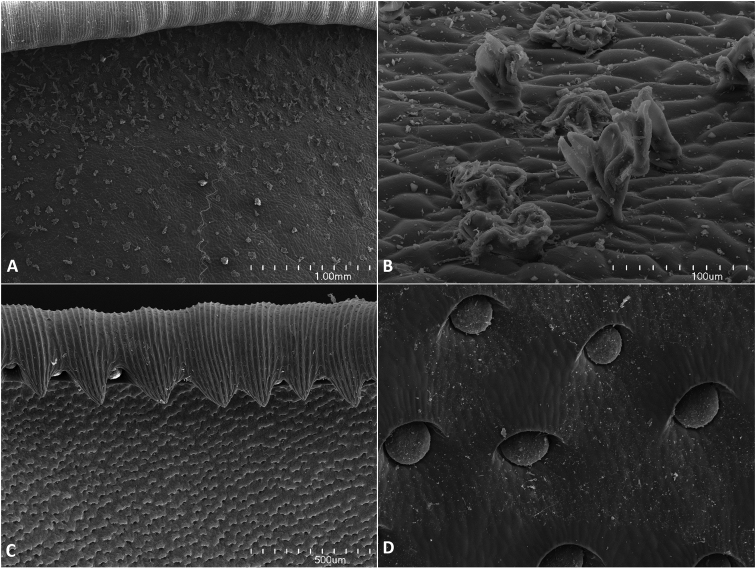
*Nepenthespudica*, SEM images of lower pitcher **A** outer wall with outer margin of peristome **B** detail of trichome on the outer wall **C** inner wall and inner margin of peristome showing eglandular zone covered with lunate cells and peristome teeth with peristomal glands **D** inner wall showing glandular zone with digestive glands. SEMs by B.J. Płachno.

**Figure 5.**
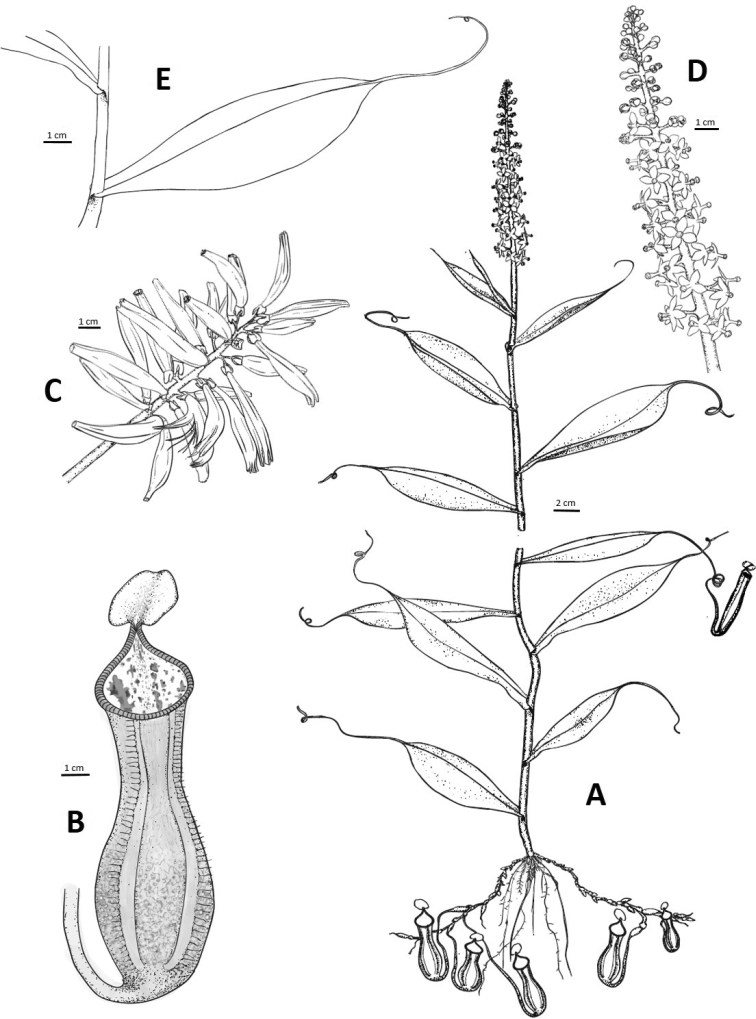
*Nepenthespudica***A** habit **B** lower pitcher **C** infructescence **D** male inflorescence **E** detail of climbing stem with a leaf. Drawn by Kateřina Janošíková.

Figs 1 , 2 , 3 , 4 , 5

#### Diagnosis.

*Nepenthespudica* differs from *N.hispida* Beck in producing short basal underground (vs. aboveground) shoots; ± glabrous (vs. hairy) stems; petiolate (vs. sessile) climbing shoot leaves with auriculate, shortly decurrent (vs. decurrent-amplexicaul) bases; rare (vs. common) upper pitchers; red (vs. green or red blotched) lower pitchers; ± glabrous (vs. hairy) mature pitchers; ventricose (vs. ovoid-ellipsoid) lower pitchers; infundibular (vs. subcylindrical, tapering) upper half of the lower pitcher; 3–5.5 cm (vs. 1.5–3 cm) wide lower pitchers; male flowers in pairs (vs. single or rarely in pairs) and androphore c. 4 mm (vs. 1.5–2 mm) long.

#### Type.

Indonesia. North Kalimantan: Malinau Regency, c. 1110 m a.s.l., 2 February 2012, *W. Tjiasmanto*, *M. Paris & M. Dančák s.n.* (BO, holotype BO1985840, isotype BO1985839).

#### Description.

Terrestrial climber producing climbing shoot and underground basal shoots. ***Climbing shoots*** up to c. 20 m long, stem glabrous, c. 4–6 mm thick, internodes c. 4 cm long. ***Underground basal shoots*** short, with reduced, partially or completely achlorophyllous leaves (nanophylls) bearing well-developed lower pitchers, not observed to branch or develop roots. ***Rosette leaves*** chartaceous, subsessile to shortly petiolate, oblanceolate, up to 16 cm long, up to 4 cm wide, apex subobtuse or acute to acuminate, base auriculate, shortly decurrent, glabrous on both sides but densely hairy with short brown hairs on the margins, tendril up to 16 cm long, uncoiled. ***Leaves of climbing shoots*** coriaceous, shortly petiolate, oblanceolate, up to 20 cm long, up to 4.5 cm wide, with 2–4 inconspicuous longitudinal veins on each side of the midrib, apex acute, base auriculate, shortly decurrent, glabrous on both sides, margins glabrous, tendril coiling. ***Rosette pitchers*** produced only briefly on aboveground rosettes, up to 9 cm high, up to 3 cm wide, thin-chartaceous, subcylindrical to ovoid in the lower part. ***Lower pitchers*** produced exclusivel on underground basal shoots, 7–11 cm high, 3–5.5 cm wide, thin-coriaceous, becoming thicker-walled and markedly sturdier when produced at depth, arising abruptly from the uncoiled tendril, ventricose, broadly ovoid to globose in the lower half, infundibular above, clearly widening towards the mouth; eglandular zone of the inner surfaces extending from the mouth almost to the middle of the pitcher; inner surface near the mouth white, conspicuously red blotched, outer surface red-purple, faintly blotched, occasionally entirely off-white when produced at depth; two fringed wings running from the bottom of the pitcher to the mouth at the front; mouth round, rising at the rear into a short neck; peristome cylindrical in section, up to 2 mm wide, inner surface with distinct teeth up to 0.8 mm long, ribs up to 0.5 mm apart, up to 0.2 mm wide; lid broadly ovate, c. 20–30 mm long, c. 20 mm wide, with short spur; large, craterlike nectar glands ± elliptic in outline, up to 0.35 mm long, scattered densely in the middle of the lower surface. ***Upper pitchers*** rarely produced, up to 9 cm high, up to 2 cm wide, thin-coriaceous, arising gradually from the tendril, narrowly infundibular at the base, subcylindrical above; eglandular zone of the inner surfaces covering upper 1/3 of the pitcher; outer surface green, inner surface near the mouth yellowish; two fringed wings running from the middle of the pitcher to the mouth at the front; mouth round, with or without very short neck; peristome cylindrical, up to 1.5 mm wide, inner surface with very short teeth, ribs up to 0.25 mm apart, c. 0.1 mm wide; lid broadly ovate, 11–16 mm long, 9–13 mm wide, with curved spur; craterlike nectar glands as in lower pitchers, up to 0.3 mm long. ***Male inflorescence*** a raceme, peduncle c. 14 cm, rachis c. 13 cm, partial peduncles 2-flowered, bracts absent, pedicels 4–7 mm long, tepals elliptic, up to 6 by 3 mm; androphore c. 4 mm long, anther head 2.5 by 1.5 mm. ***Female inflorescence*** unknown. ***Infructescence*** racemose. ***Fruit*** a fusiform capsule, reddish brown at maturity, conspicuously glossy, valves of fruits c. 45 by 4 mm. ***Seeds*** 20–25 mm long.

#### Habitat and ecology.

The species occurs on ridgetops over sandstone rocks in lower montane rainforest. The known elevational range is 1100–1300 m a.s.l. The plants frequently grow near trees whose branched roots form cavities covered with a moss layer. Lower pitchers are then copiously produced inside these cavities. If no cavities are available, the pitchers are produced directly in soil, deep litter or under moss cushions. At some sites, *Nepenthestentaculata* Hook.f. and *N.stenophylla* Mast. grew sympatrically with *N.pudica*, while a species from the *N.fusca* species complex was spotted growing epiphytically in at least one locality.

The subterranean growth habit of *Nepenthespudica* was consistently observed across the five studied sites but was not shared by the sympatric *Nepenthes* species, demonstrating that it was not simply the result of unusual local conditions. The underground shoots of *N.pudica* had no obstacles preventing them from growing upwards, suggesting that they are not negatively gravitropic as is typical of stems. Neither did they show signs of growing towards light, even when concealed only under a soft moss cushion or already slightly chlorophyllous (Fig. 2B). Based on this and their generally lateral character, it might be supposed that they are negatively phototropic rather than positively gravitropic.

#### Distribution.

The species is known only from a few adjoining localities in the western part of the Mentarang Hulu district of North Kalimantan, Indonesia. The exact locations have been withheld in order to prevent poaching by unscrupulous commercial collectors.

#### Etymology.

The specific epithet *pudica* (bashful, shy), is a feminine adjective and alludes to the fact that lower pitchers remain concealed from direct view.

**Table 1.** Main morphological differences between *Nepenthespudica* and related species, including *N.leptochila*, which is not recognised by most researchers. The characters that best differentiate *N.pudica* from the other species are in bold.

Characteristic	*N.hirsuta*	*N.hispida*	*N.pudica*	*N.leptochila*
short basal shoots	**aboveground**	**aboveground**	**underground**	**aboveground**
stem indumentum	**hairy**	**hairy**	±**glabrous**	±glabrous
stem colour	brown	purplish grey	brownish green to purplish	reddish
climbing shoot leaf shape	obovate	oblanceolate to oblong	oblanceolate	obovate-lanceolate
climbing shoot leaf width	3–6 cm	1.8–3.3	up to 4.5 cm	2.5–5.5 cm
climbing shoot leaf	petiolate	**sessile**	**petiolate**	shortly petiolate
climbing shoot leaf base	**semi-amplexicaul**	**decurrent-amplexicaul**	**auriculate, shortly decurrent**	auriculate, hardly decurrent
climbing shoot leaf texture	thin-coriaceous	thin-coriaceous	coriaceous	**chartaceous**
climbing shoot leaf apex	acute or rounded	acuminate to obtuse	acute	acute, obtuse or rounded
longitudinal veins	3–4	3	2–4 not prominent	5
tendril indumentum	hairy	hairy	hairy or glabrous	glabrous?
upper pitchers	**few**	**common**	**rare**	**present**
lower pitcher colour	**green**	**green or red blotched**	**red**	?
adult pitcher indumentum	**hairy**	**hairy**	±**glabrous**	glabrous?
lower pitcher shape	**ovoid**	**ovoid-ellipsoid**	**ventricose**	**ovoid-ellipsoid**
lower half of lower pitcher	ovoid	ovoid-ellipsoid	ovoid to globose	ovoid to globose
upper half of lower pitcher	**conical**	**subcylindrical, tapering**	**infundibular**	~**cylindrical, tapering**
lower pitcher length	up to 15 cm	5–8.5 cm	7–11 cm	up to 8 cm
lower pitcher width	up to 7 cm	**1.5–3 cm**	**3–5.5 cm**	**up to 3 cm**
eglandular zone	**almost absent**	nearly 1/2 of the surface	**nearly 1/2 of the surface**	**1/3 of the surface**
peristome width	up to 6 mm	0.5–1.2 mm	up to 2 mm	up to 1.5 mm
peristome in section	cylindrical or flattened	cylindrical	cylindrical	cylindrical or flattened
male flowers	in pairs	**single or rarely in pairs**	**in pairs**	?
androphore length	3.5–6 mm	**1.5–2 mm**	~**4 mm**	?
ecology	ridgetops	heath forest	ridgetops	?
elevational distribution	**0–1000 m**	**100–800 m**	**1100–1300 m**	~**300 m**

#### Conservation status.

*Nepenthespudica* is endemic to Borneo. It is known from five closely situated sites, which represent a single location (IUCN 2022). Both the extent of occurrence (EOO) and minimal area of occupancy (AOO) of *N.pudica* are estimated to be less than 4 km^2^. There is uncertainty as to whether the species occurs within Kayan Mentarang National Park, as its borders were not marked in the field at the time of discovery. However, the available maps suggest all the sites are actually located outside the national park, thus legally unprotected. Due to its restricted distribution, small population size and possible habitat loss, the species qualifies to be assigned preliminary conservation status as critically endangered (CR), based on criteria B1 ab(iii) and D of the IUCN Red List categories and criteria (IUCN 2012).

#### Prey composition and infauna.

We found 1785 invertebrate individuals belonging to 40 different taxa (Tables 2, 3) in suspensions sampled from five underground pitchers (found in a tree-root cavity) and one aerial rosette pitcher (growing 2 metres above the soil surface and arising from an offshoot of a fallen climbing stem). Necromass of the prey consisted of sclerites of highly digested invertebrates. It contained mainly litter- and soil-inhabiting species as well as a large amount of plant detritus. Among soil- and litter-inhabiting species we observed mites (mostly from the family Oribatidae), leaf litter–inhabiting beetles (families Scydmaenidae, Pselaphidae, Liodidae, Carabidae) and a single ant of the genus *Anochetus* (subfamily Ponerinae). These taxa are mostly mycophagous, detritophagous, or predators. However, the main and the essential prey component was a species of ant from the subfamily Myrmicinae, probably a species of the genus *Crematogaster*, which is closely associated with *Nepenthes* (Bonhomme et al. 2011). A number of individuals of an ant from the genus *Polyrhachis* were found in the aboveground rosette pitcher in contrast with their rare occurrence in underground traps.

**Table 2.** Prey composition of *Nepenthespudica* based on analysis of five underground pitchers and one aerial pitcher.

Prey composition in traps	traps from root cavity					abovegr.	total
	trap 1	trap 2	trap 3	trap 4	trap 5	trap 6	
Acarina, Oribatidae spp.	1	3	14	c. 100	20		c. 138
Acarina div.	1	1	25				27
Araneae, cf. Lycosidae	1						1
Araneae: cf. Dysderidae	1						1
Araneae					1		1
Arachnoidea, g. sp.		1	1				2
Coleoptera, Aphodiidae g. sp.	1						1
Coleoptera, Carabidae g. sp.				2			2
Coleoptera, cf. Leiodidae				6			6
Coleoptera, Pselaphidae g. sp.		1					1
Coleoptera, Scydmaenidae g. sp.	2	7	4	2			15
Coleoptera, g. sp. 1					3		3
Coleoptera, g. sp. 2			2				2
Diptera, Phoridae g. sp.			1				1
Diptera, Nematocera g. sp.		2		3			5
Diptera, g. sp.					1		1
Hemiptera, Derbidae g. sp.				1			1
Hymenoptera, Chalcidoidea g. sp.			1				1
Hymenoptera, Formicinae: Camponotuscf.gigas						4	4
Hymenoptera, Formicinae: *Polyrhachis* sp.		3	1		1	17	22
Hymenoptera, Formicinae g. sp.					3	1	4
Hymenoptera, Myrmicinae g. sp. 1	c. 500	11	c. 100	c. 50	c. 700		c. 1361
Hymenoptera, Myrmicinae g. sp. 2		1	1		25		27
Hymenoptera, Ponerinae: *Anochetus* sp.			1				1
Hymenoptera, Sphecidae g. sp.	2	1					3
**Sum of individuals**	**c. 509**	**31**	**c. 151**	**c. 164**	**c. 754**	**22**	**c. 1631**
**Sum of taxa**	**8**	**10**	**11**	**7**	**8**	**3**	**25**

Surprisingly, we found relatively numerous infauna, especially larvae of mosquitoes, nematodes and annelids in both aboveground and underground pitchers (Table 3). We identified three species of mosquitoes from two genera, *Uranotaenia* and *Culex*. Identified nematodes belong to seven families: Aphelenchoididae, Cephalobidae, Diplogastridae, Panagrolaimidae, Plectidae, Rhabditidae (dauer larvae) and Wilsonematidae. The most abundant were members of families Rhabditidae and Diplogastridae detected in the aboveground trap, which were previously recorded from the pitcher fluid of *Nepenthesmirabilis* (Lour.) Druce (Bert et al. 2011). In underground traps, nematodes were rare and in different compositions compared to the aboveground trap. The most abundant were members of the families Cephalobidae (*Heterocephalobus*) and Panagrolaimidae (*Panagrolaimus*). One of the most interesting inquilines found in the underground pitchers was a new species of annelid worm, *Pristinaarmata* (family Naididae), described previously by Schenková and Čermák (2013).

**Table 3.** Infauna composition of *Nepenthespudica* based on analysis of five underground pitchers and one aerial pitcher. (abovegr. = aerial pitcher; L1, L2, L3, L4 – larval stages).

Infauna composition in traps	traps from root cavity					abovegr.	total
	trap 1	trap 2	trap 3	trap 4	trap 5	trap 6	
Diptera, Stratiomyidae (larvae)	1				6		7
Diptera, Culicidae: *Uranotaenia* sp. 1	2 L1,1 L3,4 L4				4 L3,11 L4		22
Diptera, Culicidae: *Uranotaenia* sp. 2		9 L3	5 L2,1 L3,2 L4	1 L1,3 L2,7 L4			28
Diptera, Culicidae: *Culex* sp.	4 L4	4 L2,1 L4	3 L4	1 L4			13
Diptera, Acalyptrata			2 L2		1 L1, 4 L2	8 L1	15
Annelida, Naididae: *Pristinaarmata*				6			6
Nematoda, Cephalobidae: *Heterocephalobus* sp.	8						8
Nematoda, Aphelenchida: *Aphelenchoides* sp. 1				1			1
Nematoda, Aphelenchida: *Aphelenchoides* sp. 2				1			1
Nematoda, Panagrolaimidae: *Propanagrolaimus* sp.				8			8
Nematoda, Wilsonematinae: *Ereptonema* sp.				1			1
Nematoda, Plectidae: *Plectus* sp.				1			1
Nematoda, Diplogasteridae: *Pristionchus* sp.						27	27
Nematoda, Rhabditidae (dauer larvae)						16	16
**Sum of individuals**	**20**	**14**	**13**	**30**	**26**	**51**	**154**
**Sum of taxa**	**4**	**2**	**3**	**8**	**3**	**3**	**14**

#### Selected specimens examined.

See Suppl. material 1.

## ﻿Discussion

*Nepenthespudica* is the first carnivorous species confirmed to use pitfall traps specifically in the subterranean environment. It produces almost exclusively underground pitchers that are well developed and fully functional. Although in some species of *Nepenthes* pitchers are occasionally reported to develop in plant litter or directly in the soil (Salmon 1993; Nerz et al. 1998; Clarke 2001; Ghazalli et al. 2020), no species that specifically targets this environment to this extent has been documented to date. This is not surprising, as pitchers are generally much larger than other types of traps and are rather fragile due to their hollow character. Therefore, they are generally unsuitable for the soil environment, where considerable pressure is needed to form a cavity. As the pitchers of *N.pudica* are of a typical size for the genus, they are by far the biggest underground traps among all known carnivorous plants. While the other genera of carnivorous plants that produce underground traps are, due to the small size of their traps, capable of catching only microscopic or very small prey (Seine et al. 2002; Pereira et al. 2012), the pitchers of *N.pudica* catch prey of the same size as other pitcher plants.

The traps of carnivorous plants are complex and metabolically costly organs that must be produced at the expense of tissues optimised for photosynthesis (Givnish et al. 1984; Pavlovič and Saganová 2015). In pitcher plants, this trade-off often manifests in the separation of primary prey- and light-harvesting structures spatially–e.g. on an intra-leaf level as in most *Nepenthes*—and also temporally, as in the seasonal production of solely photosynthetic leaves by *Cephalotus* Labill. and some *Sarracenia* L., both examples of separation on an inter-leaf but intra-shoot level (McPherson and Schnell 2011; Cross et al. 2019). In *N.pudica*, this ‘division of labour’ is unusually displayed at the level of the shoots. This strategy is analogous to that of certain strongly shoot-dimorphic aquatic *Utricularia*, such as *U.intermedia* Hayne, whose specialised carnivorous shoots penetrate a loose organic sediment while the green stems seek sunlight in clear water near the surface (Adamec 2007).

Each leaf of a typical *Nepenthes* comprises an entirely photosynthetic lamina-like phyllodium and a predominantly carnivorous and only marginally photosynthetic pitcher (Pavlovič et al. 2007, 2009; Karagatzides and Ellison 2009). The unusual architecture of *N.pudica* (Fig. 5A) appears to have largely freed it from the phylogenetic constraint of having functional phyllodia and pitchers in close physical proximity, and thereby allowed it to exploit a novel source of prey in the form of the subterranean environment, limiting competition with sympatric congeners. However, this body plan is likely to come with certain costs. Subterranean pitchers, by virtue of having to displace surrounding substrate as they grow, might be expected to have significantly thicker walls and a higher concentration of structural compounds (e.g. lignin) than those produced above ground. Preliminary observations indicate that underground pitchers are indeed markedly thicker-walled and sturdier (M. Dančák & M. Golos, pers. observ.). All else being equal, this would increase their construction costs, partly offsetting benefits from carnivory, and likely dictate longer pitcher lifespans, reflecting a greater ‘payback time’ for recovery of these costs (see Osunkoya et al. 2008). And this does not even consider the additional stem biomass needed for dimorphic shoots. Moreover, the greater separation of the two types of assimilatory organs in *N.pudica* must presumably necessitate two-way exchange of nutrients and photosynthates over much greater mean distances than in species with typical pitcher–phyllodium pairs (see Osunkoya et al. 2007). All told, the benefit from subterranean carnivory must be significant to make up for these additional costs and this is perhaps the reason this strategy is not seen more widely across the genus.

Among *Nepenthes*, the species that come closest to this degree of shoot specialisation are perhaps those in which pitchers produced in low-light conditions near ground level are borne on crowded, greatly reduced phyllodia (the latter sometimes termed ‘nanophylls’; Cheek 2015). The best known of these, *N.ampullaria* Jack, additionally produces largely or entirely pitcherless climbing stems (Tan and Wong 1996), mirroring the situation in *N.pudica*, though the latter’s production of solely carnivorous shoots appears to be unique among *Nepenthes* and indeed among all pitcher plants. Also of note is the comparatively little-known *N.rhombicaulis* Sh.Kurata of Sumatra, which rarely if ever produces upper pitchers and has been speculated to target underground prey, though until now its lower pitchers have only been documented to develop within dense moss and detritus rather than being truly subterranean (Salmon 1993; Schmid-Hollinger 1994; Clarke 1997a, 2001). This species, which appears to occupy a similar ecological niche to members of the *N.hirsuta* group in Borneo, would be a prime candidate for further investigation in this regard.

**Figure 6.**
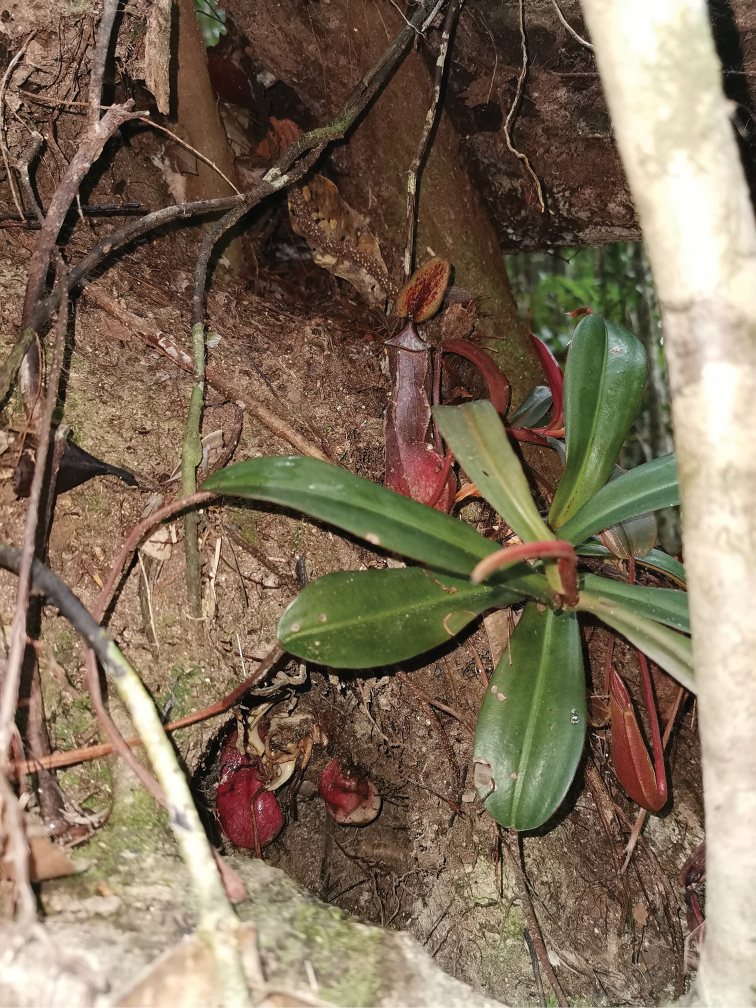
*Nepenthes* sp. with excavated underground traps (bottom left) from a locality in the Berau region of East Kalimantan. Photograph by M.R. Golos.

Since the discovery of *Nepenthespudica*, field observations in the Berau region of East Kalimantan (M. Golos, pers. observ. June 2019) have revealed a similar taxon that likewise produces achlorophyllous subterranean shoots bearing nanophylls with reddish pitchers (Fig. 6). This taxon also produces few aerial traps, though it notably differs from the type population of *N.pudica* in growing at considerably lower elevations. Its precise taxonomic affinities have yet to be determined.

As was demonstrated above, the prey of *Nepenthespudica* consists of various species of soil- and litter-inhabiting fauna. With 25 different taxa, the diversity of identified prey was rather high, which is typical for species growing at higher elevations (Adam 1997). However, ants were the main prey component found in both aerial (subfamily Formicinae) and lower pitchers (subfamily Myrmicinae). At this point, we can assume that *N.pudica* is predominantly an ant specialist, as are the majority of *Nepenthes* species.

Consistently with other *Nepenthes* species, *N.pudica* harbours relatively numerous and diverse infauna in both types of pitchers (154 individuals and 14 identified taxa). Besides mosquitoes, which are commonly associated with pitcher plants (Vong et al. 2021), larvae of aquatic Diptera (family Stratiomyidae and subsection Acalyptrata) were detected as well. The insect-trapping structures of pitcher plants (especially Nepenthaceae and Sarraceniaceae) frequently harbour dipteran larvae, which utilize the food niche in pitchers (Adlassnig et al. 2011). Members of the family Stratiomyidae are true aquatic organisms inhabiting many kinds of phytotelmata such as tree holes, leaf axils and modified leaves (Greeney 2000); however, their presence in pitchers is not as common in comparison with members of other dipteran families such as Syrphidae, Ceratopogonidae or Chiromidae (Kitching 2000). Rather surprising is the fact that lower pitchers of *N.pudica* also contained abundant dipteran infauna, including mosquitoes. This indicates that the tree-root cavities from which samples were taken were accessible to the outside-living invertebrates. Therefore, even the hidden lower pitchers can serve as a stable and permanent water habitat (phytotelma) similar to other *Nepenthes* species or other plants, e.g. unrelated Bromeliaceae (Thorp and Rogers 2015), and play an essential role in the development of these symbionts, especially during dry periods. However, underground pitchers produced in compacted substrate (Fig. 2A) would presumably not be similarly accessible to ovipositing insects.

Nematodes formed the other large group of infauna. Identified individuals belonged to families Aphelenchoididae, Cephalobidae, Diplogastridae, Panagrolaimidae, Plectidae, Rhabditidae (dauer larvae) and Wilsonematidae. The most abundant were members of the genus *Pristionchus* (Diplogastridae), detected only in the aboveground trap and obviously associated with the main prey, an ant species of the genus *Polyrhachis*. Species of the genus *Pristionchus* feed selectively on bacteria and fungi decomposing insect carcasses (Rae et al. 2008), including various genera of ants, e.g. *Formica*, *Lasius* and *Myrmica* (Wahab 1962; Ishaq et al. 2021). The nematodes detected in lower pitchers were members of genera generally living in soil and water environments and feeding on bacteria and fungi decomposing organic material (Bongers 1990). The only exception was the genus *Halicephalobus*, the species of which are aquatic but occur in extreme environments (Borgonie et al. 2011; Geraert et al. 1988), various phytotelmata (Andrassy 1952; Körner 1954) or as parasites (Stefanski 1954).

Probably the most interesting species living in the pitchers of *Nepenthespudica* was the annelid worm *Pristinaarmata* (Naididae), which was described from and found so far only in its lower pitchers. For the description and discussion on its relation to *N.pudica*, see Schenková and Čermák (2013).

The living strategy of *Nepenthespudica* can be viewed as an advantageous evolutionary adaptation. As carnivorous plants are highly dependent on prey for organic nutrients essential for reproductive success (Zamora et al. 1997), strong selective pressures may have acted on traits related to prey capture (Ellison et al. 2001). Hence, the potentially strong competition for prey and possible environmental limitations in the forest understorey (e.g. dryness affecting ridgetops) might be avoided by moving the traps underground.

*Nepenthespudica* belongs to the *N.hirsuta* group, which is endemic to Borneo and includes at least two putative close relatives: *N.hirsuta* Hook.f. and *N.hispida*. Another two species are sometimes considered members of this group, namely the Bornean *N.macrovulgaris* J.R.Turnbull & A.T.Middleton and *N.philippinensis* Macfarl. from the island of Palawan (Cheek and Jebb 1999). However, the recent phylogeny of the genus (Murphy et al. 2020), while proving the close relationships of *N.hirsuta* and *N.hispida*, does not support the close affinities of *N.macrovulgaris* and *N.philippinensis*, either mutually or to *N.hirsuta* and *N.hispida*. *Nepentheshirsuta* and *N.hispida* share a combination of traits that distinguishes this group from the rest of the genus. These are especially the growth form (well-developed rosetted, non-climbing phase), hairy stem, more or less ovoid shape of the lower pitchers, oblique pitcher mouth, ± cylindrical peristome, lid without appendages and flowers usually in pairs (Cheek and Jebb 1999). *Nepenthespudica*, while possessing most of these characteristics, shows several unique traits. These are namely a) underground basal shoots (the other species form aboveground basal shoots); b) upper pitchers are only rarely produced in lower parts of the climbing stem; c) lower pitchers are produced exclusively underground; d) the shape of the lower pitchers is ventricose with the lower half ovoid to globose and the upper half infundibular. Another possible member of the *N.hirsuta* group, *Nepenthesleptochila* Danser, was described from northern North Kalimantan (Mt. Djempanga; Danser 1928), but this name is usually considered a heterotypic synonym of *N.hirsuta* (Clarke 1997b; Jebb and Cheek 1997; Cheek and Jebb 2001; Phillipps et al. 2008; McPherson et al. 2009). Nevertheless, the original description mentions several significant differences compared to *N.hirsuta* (e.g. a well-developed eglandular zone inside the pitchers, glabrous stems and pitchers, and much smaller pitchers) so its identity is at least questionable. *Nepenthesleptochila* also bears considerable resemblance to *N.pudica*, especially in being rather glabrous. However, the two taxa differ in all the four previously mentioned characters typical for *N.pudica* and therefore we do not consider them conspecific. For a comparison of critical diagnostic characters of *N.hirsuta* (excluding *N.leptochila*), *N.hispida*, *N.leptochila* and *N.pudica*, see Table 1.

## Supplementary Material

XML Treatment for Nepenthes pudica

## Supplementary materials

Supplementary material 1 List of examined specimens Data type docx file Explanation note *Nepentheshirsuta* (including *N.leptochila*) and *Nepentheshispida.* https://binary.pensoft.net/file/704713This dataset is made available under the Open Database License (http://opendatacommons.org/licenses/odbl/1.0/). The Open Database License (ODbL) is a license agreement intended to allow users to freely share, modify, and use this Dataset while maintaining this same freedom for others, provided that the original source and author(s) are credited.Martin Dančák, Ľuboš Majeský, Václav Čermák, Michal R. Golos, Bartosz J. Płachno, Wewin Tjiasmanto
