# Supplementary material for: ﻿First record of functional underground traps in a pitcher plant: Nepenthespudica (Nepenthaceae), a new species from North Kalimantan, Borneo
Source: PhytoKeys. 2022 Jun 23;201:77–97. doi: 10.3897/phytokeys.201.82872 (PMC9848998; doi:10.3897/phytokeys.201.82872)
Supplement: Supplementary material 1 — List of examined specimens [file phytokeys-201-077_article-82872__-s001.docx]

**Supplementary material**

List of examined specimens

***Nepenthes hirsuta*** (including *N. leptochila*)—BRUNEI. **Temburong:** Ulu Ropan – Belalong watershed, 2000–2500 ft [≈610–760 m] a.s.l., 12 February 1959, *(Ashton) BRUN 5238* (BRUN, K! [2 sheets]) [pitcherless climbing stem with infructescence (sheet 1), pitcherless climbing stem with female inflorescence (sheet 2); “Mor soil on clay ridge.”]; Bukit Belalong, north ridge, no elevation data, 21 July 1989, *Wong 1447* (BRUN, K!) [separate climbing stems with upper pitchers and male inflorescences]; Ridge on upstream side of the Sungai Temburong Machang, [r]idge top, 320 m a.s.l., 22 August 1990, *Wong 1998* (BRUN, K!) [stem with lower pitchers]; Summit of Bukit Belalong, no elevation data, 27 February 1991, *Argent & Pendry 91155* (E, L!) [climbing stem with pitcher, separate stem with male inflorescences; “Open area at edge of helicopter pad”]; Batu Apoi Forest Reserve, [s]ummit ridge of Bukit Belalong, [s]ubcamp on the Belalong ridge, 4°33’N 115°09’E, 980 m a.s.l., 13 March 1992, *Nielsen 1077* (AAU, K!) [stem with lower pitchers; “setap shale formation”]; Amo, Bukit Tudal, 4°23’N 115°18’E, 1060 m a.s.l., 4 October 1994, *(Said et al.) BRUN 15805* (BRUN, K!) [offshoot with lower pitchers; “Granite [geology] Low montane forest”]. **Tutong:**Rambai, Ladan Hill Forest Reserve, Bukit Bedawan, North of LP-263, 4°29’33”N 114°48’52”E, 529 m a.s.l., 25 March 1997, *(Kalat et al.) BRUN 18054* (BRUN, K!) [climbing stem with upper pitcher; “Sandy soil with humus over sandstone. Mixed dipterocarp forest. On the ridge.”].

INDONESIA. **Central Kalimantan:** P.B.U. [Project Barito Ulu] base camp and environs, [t]rail lake to waterfall along river, approx. 0.02°S 114.06°E, elevation given as “0”, 11 June 1990, *Ridsdale PBU484A* (L! [2 sheets]) [pitcherless climbing stem with female inflorescence (both sheets); “*Tristania*–*Gymnostoma* heath forest”]; Barito Ulu, 0°02’S 114°06’E, 140 m a.s.l., 8 August 2005, *Mansur 38* (BO!) [stem with lower pitcher; “Kerangas Forest”]. **East Kalimantan:** West Kutei, Mt. Palimasan near Tabang on Belajan River, 500 m a.s.l., 16 September 1956, *Kostermans 13099* (BO! [2 sheets], K!, L) [BO—climbing stem with female inflorescence and infructescence (sheet 1), climbing stem with upper pitcher (sheet 2), K—pitcherless climbing stem with infructescence; “*Agathis* forest on acid, sandy, waterlogged soil.”]; Gunong Batukenye, northwest of Tabang, 150–550 m a.s.l., 9 January 1979, *Murata, Iwatsuki, Kato & Mogea B-1754* (BO!) [stem with male inflorescences; “In tropical rain forest”]; Gunong Mendam, north of Tabang, 300–880 m a.s.l., 15 January 1979, *Murata, Iwatsuki, Kato & Mogea B-2617* (BO!) [stems with lower pitchers; “In evergreen forest”]; Mittellauf des Mah[a]kam [middle reaches of the Mahakam], 100 m (?) a.s.l., 20 May 1981, *Schmutz 4915* (L!) [separate leaves with lower pitchers; “Primärwald, abgründig [primary forest, sloping]”]; Long Iram subdistrict, Maruwai, North East Lampunut, 0°30’S 114°55’E, 271 m a.s.l., 7 March 1999, *Kessler et al. PK 2438* (L!) [rosette with lower pitchers; “Inland Kerangas forest, called by Dayak Bakumpai people ‘Perenget’, often waterlogged. Vegetation on white cr[y]stalline sand or sandstone.”]; Long Iram subdistrict, Maruwai, North East Lampunut, 0°30’S 114°55’E, 290 m a.s.l., 8 March 1999, *Kessler et al. PK 2464* (L! [2 sheets]) [climbing stem with upper pitchers (sheet 1), stem with offshoots bearing lower pitchers (sheet 2); “Primary forest, very humid, near stream, alluvial soil.”]. **North Kalimantan:** G. Djempanga, no elevation data, September 1912, *Amdjah 730* (lecto BO, iso BO, K! [2 sheets]) [pitcherless climbing stem (sheet 1), rosette with lower pitcher (sheet 2); lectotype of *N. leptochila*]; Tarakan, oilfield Sesanip, “low” elevation, 13 December 1953, *Meijer 2480* (BO!, L), *2480a* (BO! [2 sheets], L), *2485* (BO!, L), *2486a* (BO! [2 sheets], K!, L!), *2489a* (BO!) [*2480*—climbing stem with pitchers, *2480a*—stem with lower pitchers (both sheets), *2485*—climbing stem with upper pitchers and male inflorescences, *2486a*—stem with lower pitcher (BO; both sheets), climbing stem with upper pitcher(s) (K, L), *2489a*—stem with lower pitchers; “forest margin, sandy soil”]; Tarakan, oilfield Djuatan – G. Tjangkol, no elevation data, 15 December 1953, *Meijer 2550* (A, BO!, K!, L, SING) [BO—stem with lower pitchers, K—climbing stem with upper pitcher; “*Agathis*-forest”]; Tarakan, [f]orest near kg. 4, “low” elevation, 17 December 1953, *Meijer 2593* (BO! [2 sheets], K!, L) [BO (sheet 1), K—climbing stem with upper pitcher and male inflorescences, BO (sheet 2)—climbing stem with upper pitcher; “Primary forest”]. **West Kalimantan:** Goenoeng Damoes, no elevation data, 22 October 1893, *Hallier B642* (BO! [2 sheets], L) [separate lower pitchers and stems (both sheets)]; Goenoeng Damoes, no elevation data, 22–23 October 1893, *Hallier B644* (BO!, L) [climbing stems and upper pitcher]; [Mt.] Liang [G]agang, no elevation data, 7–14 March 1894, *Hallier s.n.* (K!) [separate leaves and upper pitchers]; G. Bentuang area, 5–10 km north of Masa village, 0°56’N 100°26’E, 1120 m a.s.l., 28 June – 6 July 1989, *Burley, Tukirin et al. 3051* (A, BO, L!) [stem with pitcher; “Mixed dipterocarp forest. Ridge area.”]; Serawai, Sungai Merah, [0].5 km to NE of camp along ridge and surrounding hills, 0°33’40”S 112°37’32.7”E, 650 m a.s.l., 13 February 1995, *Church & Mahyar 1935* (A, BO!) [climbing stem with pitcher and infructescence; “Hill Dipterocarp forest, associ[at]es include: *Shorea*, *Dipterocarpus*, *Hopea*, *Elaeocarpus*.”].

MALAYSIA. **Labuan:** Labuan I[sland], no elevation data, 1877–1878, *Burbidge s.n.* (K!) [climbing stem with upper pitchers; tentatively identified as *N. leptochila* by B.H. Danser, 1930]. **Sabah:** Keningau District, Tambulanon Keningau, 25 October 1983*, (Patrick & Kumin) SAN 68879* (L!, SAN) [climbing stem with upper pitcher and male inflorescence; “On rocky soil, Primary forest, Undulating land.”]; Sepulut, Tobou Sepulut, F.R. [Forest Reserve] Sg, no elevation data, 26 July 1984, *(Fedilis & Sumbing) SAN 106870* (K!, KEP, L, SAN, SAR) [stem with lower pitchers; “Hill top”]; Nabawan, Meliau Basin, Gn. Lotung exp., no elevation data, 24 April 1988, *(Madani) SAN 124494* (K!, SAN) [pitcherless climbing stem; “Primary forest”]; Tambunan, Gn. Trusmadi, no elevation data, 10 August 1988, *(Fidilis) SAN 125459* (K!, SAN) [pitcherless climbing stem with infructescence; “Hillsides”]; Maliau Basin, eastern edge, near escarpment, no elevation data, 15 February 1992, *Phillipps ALFB 514/92* (K! [2 sheets]) [stems with lower pitchers (both sheets); “Montane mixed forest with *Agathis* + *Casuarina*. Sandstone soils.”]; Maliau Basin, eastern escarpment rim, c. 1200 m a.s.l., 15 February 1992, *Lamb ALFB 516/92* (K! [2 sheets]) [stems with dissected lower pitchers (both sheets); “Mixed montane forest on sandstone ridge”]; Lahad Datu, Gunung Nicola, 850 m a.s.l., 24 February 1992, *(Kulip & Molubin) SAN 133347* (K! [2 sheets], SAN), *(Kulip, Donggop & Radin) SAN 133348* (K!, L!, SAN), *133350* (K! [2 sheets], SAN) [*133347*—stem with lower pitchers (sheet 1), leaf and lower pitchers (sheet 2), *133348*—stem with lower pitchers (K), separate leaves and pitchers (L), *133350*—separate leaves and lower pitchers (both sheets); “lower montane forest”]; Lahad Datu, Danum Valley, Gunung Nicola, ridge, 600–800 m a.s.l., 25 February 1992, *Lamb ALFB 554/92* (K! [2 sheets]) [rosette with lower pitchers (sheet 1), leaf with lower pitcher (sheet 2); “Stunted forest, partly mossy in places.”]; Maliau Basin, no elevation data, August 1992 (received at Kew), *Beaman 1134* (K! [2 sheets]) [rosette with lower pitchers (both sheets)]; Imbak Canyon, [n]ext to path leading up from 8-Tiered Waterfall to ridge, 5°07’N 116°57’E, 230 m a.s.l., 13 May 2004, *Clark et al. 10* (K!, KEP, SAN) [stem with lower pitchers, separate upper pitcher; “Forest transitional from Lowland Dipterocarp rainforest to Karangas”]. **Sarawak:** no further locality data, 2700 ft [≈820 m], 1857, *Lobb 92* (holo K!) [leaf with lower pitcher; holotype of *N. hirsuta* var. *glabrata*]; Lawas River, 1867, *Low s.n.* (holo K! [3 sheets]) [stem with offshoots bearing lower pitchers (sheet 1), climbing stem with upper pitcher, separate dissected pitcher (sheet 2), stem with lower pitcher (sheet 3); holotype of *N. hirsuta*]; Lawas River, 2000–3000 ft [≈610–910 m], 1877–1878, *Burbidge s.n.* (K!) [stem with lower pitchers; “shady jungle”]; Matang Mt, no elevation data, 11 August 1911, *[Unknown collector] s.n.* (BO!, SAR) [stem with lower pitchers]; Matang, 7 January 1915, *Ridley s.n.* (K! [3 sheets]) [pitcherless climbing stem with male inflorescence (sheet 1), pitcherless climbing stem with female inflorescences (sheet 2), climbing stem with infructescence, separate leaf with upper pitcher (sheet 3)]; Mount Dulit (Ulu Koyan), c. 850 m a.s.l., 21 September 1932, *“Native Collector” 537* (K! [2 sheets]) [stems with lower pitchers (both sheets); “Sand forest”; identified as *N. leptochila* by B.H. Danser, 22/12/1933]; Mount Dulit (Ulu Koyan), c. 900 m a.s.l., 3 October 1932, *Richards 2111* (K! [2 sheets]) [stem with lower pitchers (sheet 1), pitcherless climbing stem (sheet 2); “On ground in “heath” forest.”; identified as *N. leptochila* by B.H. Danser, 1933]; Division 1, Kuching, no elevation data, 15 April 1954, *Brooke 8346* (L!) [stem with lower pitchers; “Amongst young trees.”; tentatively identified as *N. leptochila* by J. Schlauer, 14/10/1997]; Gunong Pueh, 3800 ft [≈1160 m] a.s.l., 24 September 1955, *(Purseglove & Shah) P 4733* (K!, SING) [climbing stem with pitcher; “Forest”]; no further locality data, no elevation data, 4 July 1960, *Smythies 12640A* (K!) [pitcherless climbing stem with female inflorescence]; 1st Div., Gunong Aping, 2000 ft [≈610 m] a.s.l., 13 July 1961, *Collenette 720* (K!, SAR) [separate leaves with lower pitchers; “Near base of main escarpment. On shady rocks.”; identified as *N. leptochila* by M.R. Cheek, 10/1994]; 2nd Div., Sungei Lemanak, Bukit Bangai, crest of main spur, near summit, 3000 ft [≈910 m] a.s.l., 22 October 1961, *Collenette 836* (K! [2 sheets], L!, SAR), *837* (K!, SAR) [*836*—stems with lower pitchers (K; both sheets), stem with intermediate pitcher (L), *837*—climbing stem with upper pitchers and female inflorescence; “Among rocks, moss and thin trees.”]; Lundu, G. Pueh F.R. [Forest Reserve], 3500 ft [≈1070 m] a.s.l., 5 November 1961, *Smythies 15652* (K! [2 sheets], SAR) [pitcherless climbing stem with male inflorescences (sheet 1), rosette with lower pitchers (sheet 2); “Fairly common in montane forest in a narrow altitudinal zone 3,300–3,500’ [≈1010–1070 m].”]; Temiai/Temalad watershed, Ulu Mujong, Balleh, 950 m a.s.l., 21 March 1964, *(Ashton) S 13993* (K!, SAR) [climbing stem with offshoots bearing lower pitchers; “Heath forest on sandstone plateau (Kakus member, Nyabau formation)”]; Hose mountains, Ulu Tian, Carapa Lop, c. 950 m a.s.l., 27 March 1964, *(Asah ak Unyong) S 21145* (K!, SAR) [stem with lower pitchers; “Heath forest on Kakus sandstone plateau”]; Hose mountains, Mujong, Ulu Amau, Bt. Lumut, c. 900 m a.s.l., 18 April 1964, *(Ashton) S 21271* (K!, SAR) [climbing stem with upper pitcher; “Mossy Heath forest, edge of Kakus sandstone scarp”]; Santubong, Telok Pasir, c. 100 m a.s.l., 15 June 1964, *(Banying & Sibat) S 21546* (K!, SAR) [climbing stem with upper pitcher; “Heath forest, sandstone slope”]; South side of Bungoh range, near summit, no elevation data, 5 December 1969, *(Anderson, Ilias & Dawos) S 29316* (K!, SAR) [stem with lower pitcher; “Submontane Kerangas on dip slope, 300 ft. below summit ridge. On sandstone rock.”]; 5th Division, Lawas, Kota F.R. [Forest Reserve], Ulu Sg. Masia, 3600 ft [≈1100 m] a.s.l., 12 March 1973, *(Tong & Jugah) S 32921* (K!, L, SAR) [pitcherless climbing stem with male inflorescence; “On gentle slope near stream in Kerangas forest [...] on sandy soil.”]; Base camp at Bt. Sadok, no elevation data, 14 October 1982, *(Banyeng & Paie) S 45048* (K!, KEP, L, SAN, SAR) [pitcherless climbing stem with developing inflorescence; “On ridge. Mossy Forest.”]; 1st Division, vicinity of Lundu, Ulu Sungei Semantan, up to 200 m a.s.l., 20 December 1988, *Kessler 271* (L! [2 sheets], SAR) [climbing stem with infructescence (sheet 1), basal offshoot with lower pitchers and roots (sheet 2); “Kerangas forest”]; 1st Division, Mt. Mattang (Gunung Serapi), up to 200 m a.s.l., 24 December 1988, *Kessler 332* (L! [2 sheets]) [climbing stem with upper pitcher (sheet 1), pitcherless climbing stem (sheet 2); “Thin boled primary forest”]; Kapit, B[atang] Balui, B[ukit] Kumbong, 950 m a.s.l., 25 February 1992, *(Runi, Lai, Awang, Jugah et al.) S 60033* (K!, KEP, SAR) [stem with lower pitcher; “Upper Mixed Dipterocarp Forest.”]; Marudi, Sungai Silat Basin, SW ridge of Bukit Palutan, 2°48’N 115°01’53”E, 1100 m a.s.l., 28 March 2003, *(Lim & Lee) S 90471* (KEP, L!, SAN, SAR, SING) [rosette with lower pitcher; “Mossy forest on sandstone ridge.”]. **Uncertain locality:** North Borneo, no further locality data, no elevation data, 1877–1878, *Burbidge s.n.* (K! [2 sheets]) [stems with lower pitcher(s) (both sheets); identified as *N. hirsuta* var. *glabrescens* by J.M. Macfarlane, 1908].

***Nepenthes hispida***—BRUNEI. **Belait:** Teraja, path to Sarawak border, c. 4°17’N 114°25’E, 5 m a.s.l., 25 December 1991, *De Vogel 9435* (L!) [stem with lower pitcher; “Peat swamp forest 25 m high, with dense undergrowth, on thick water-logged layer of peat, much pole trees. Alluvial flat of Baram river.”].

MALAYSIA. **Sarawak:** Lawas [River], 2000–3000 ft [≈610–910 m], 1877, *Burbidge s.n.* (lecto W, iso BM! [2 sheets], K, W [2 sheets]) [stems with lower pitchers (both sheets at BM); “No flowering or seedling specimen found.”; lectotype of *N. hispida*]. **Uncertain locality:** North Borneo, no further locality data, no elevation data, 1877–1878, *Burbidge s.n.* (US! [4 sheets]) [stems with lower pitchers (all sheets)].
